# A Modified Lysimeter Study for Phyto-Treatment of Moderately Saline Wastewater Using Plant-Derived Filter Bedding Materials

**DOI:** 10.3389/fmicb.2021.767132

**Published:** 2021-12-06

**Authors:** Deepak Marathe, Karthik Raghunathan, Anshika Singh, Prashant Thawale, Kanchan Kumari

**Affiliations:** ^1^CSIR-National Environmental Engineering Research Institute (CSIR-NEERI), Nagpur, India; ^2^Academy of Scientific and Innovative Research (AcSIR), Ghaziabad, India; ^3^CSIR-National Environmental Engineering Research Institute (CSIR-NEERI), Kolkata Zonal Centre, Kolkata, India

**Keywords:** lysimeter, total dissolved solids (TDS), hydraulic loading rate, saline wastewater, filter bedding material, *Eucalyptus camaldulensis*

## Abstract

The present study focuses on determining the phyto-treatment efficiency for treatment of moderately saline wastewater using organic raw materials, such as rice husk, coconut husk, rice straw, and charcoal. The moderately saline wastewater with total dissolved solids (TDS) concentration up to 6143.33 ± 5.77 mg/L was applied to the lysimeters at the rate of 200 m^3^ ha^–1^ day^–1^ in five different lysimeter treatments planted with *Eucalyptus camaldulensis* (T1, T2, T3, T4, and T5). T1 was a control without any filter bedding material, whereas rice straw, rice husk, coconut husk, and charcoal were used as filter bedding materials in the T2, T3, T4, and T5 treatment systems, respectively. Each treatment showed significant treatment efficiency wherein T3 had the highest removal efficiency of 76.21% followed by T4 (67.57%), T5 (65.18%), T2 (46.46%), and T1 (45.5%). T3 and T4 also showed higher salt accumulation, such as sodium (Na) and potassium (K). Further, the pollution load in terms of TDS and chemical and biological oxygen demand significantly reduced from leachate in the T3 and T4 treatments in comparison with other treatments. Parameters of the soil, such as electrical conductivity, exchangeable sodium percentage, and cation exchange capacity did not show values corresponding to high salinity or sodic soils, and therefore, no adverse impact on soil was observed in the present study. Also, *Eucalyptus camaldulensis* plant species showed good response to wastewater treatment in terms of growth parameters, such as root/shoot weight and nitrogen, phosphorus, and potassium (NPK) uptake, plant height, biomass, and chlorophyll content. Root and shoot dry weight were in the order T3 (51.2 and 44.6 g)>T4 (49.3 and 43.5 g) > T5 (47.6 and 40.5 g) > T2 (46.9 and 38.2 g) > T1 (45.6 and 37.1 g). Likewise, the total chlorophyll content was highest in T3 (12.6 μg/g) followed by T4 (12.3 μg/g), T5 (11.9 μg/g), T2 (11.5 μg/g), and the control, that is, T1 (11.0 μg/g). However, the most promising results were obtained for T3 and T4 treatments in comparison with the control (T1), which implies that, among all organic raw materials, coconut and rice husks showed the highest potential for salt accumulation and thereby wastewater treatment. Conclusively, the findings of the study suggest that organic raw material–based amendments are useful in managing the high salts levels in both plants and leachates.

## Introduction

Rapid industrialization and population growth has intensified the generation of a huge amount of waste and other pollutants in the environment (Shukla et al., 2020). The present technologies are often obstructed with the inefficiencies to promote the valuable use of the waste for its valorization (Singh and Kumari, 2019). This, in turn, requires the establishment of a stringent policy framework and management practices facilitating waste conversion to wealth through its proper use thereafter owing to its sustainable management (Singh et al., 2021). Similarly, there is a huge increase in the generation of industrial effluent discharge, which is directly associated with the increase in the number of small- and large-scale industries (Edokpayi et al., 2017). Increasing environmental pollution has caused a number of environmental impacts, such as climate change (Kundzewicz and Krysanova, 2010), water pollution, water scarcity, soil contamination, biodiversity loss, etc., which have been quite evident from the last many decades (Edokpayi et al., 2017). One of the major consequences includes contamination and depletion/scarcity of natural freshwater resources. It is further predicted that about 60% of the world population will face the water scarcity problem by the year 2030 due to increasing water pollution and water scarcity (WEF-WRG, 2012; Dash et al., 2016). Such consequences have confronted the scientific communities to look for sustainable opportunities for wastewater treatment and reuse before discharging to freshwater bodies.

To manage the enormous volumes of wastewater, several innovative technologies have been deployed globally for its recycling, treatment, and reuse. However, the amount generated is far more than the amount that is treated. For instance, India generates approximately 38,000 million liters per day (MLD) of wastewater, whereas the sewage treatment capacity of the whole country is limited to only 12,000 MLD (CPCB, 2016). In fact, the available treatment technologies are not utilized efficiently due to operational and maintenance problems.

Direct land disposal of industrial effluent also poses adverse changes in soil physicochemical properties, such as soil pH, micronutrients and sodium absorption rate (SAR), and directly impacts soil fertility, seed germination, and crop growth and productivity (Chowdhary et al., 2018). Furthermore, the groundwater quality is also deteriorated due to leaching of organic and inorganic contents from industrial effluents (Mohana et al., 2009). One of the crucial parameters to be monitored during the treatment and disposal of wastewater is its total dissolved solids (TDS) level as high TDS poses major challenges, such as soil salinity and groundwater contamination. The TDS levels may vary from industry to industry, such as tannery, textile, milk, distillery, etc., and can reach up to 100,000 mg/L with biological oxygen demand (BOD) values up to 200,000 mg/L (Mortula and Shabani, 2012). Treatment and disposal of wastewater containing high TDS by conventional methods (such as reverse osmosis, membrane filtration, etc.) require complex operations and maintenance (Hareesh et al., 2017). Studies state that the available conventional wastewater treatment techniques have good efficiencies, but at the same time, they are energy intensive, Capex and Opex intensive, and require high technical skills (Zhao et al., 2019; Sahu, 2021). Therefore, to mitigate such constraints, great attention has been paid to identify sustainable and natural treatment methods, such as land-based treatment and disposal of industrial effluents or wastewater mitigating the adverse impacts of water and land pollution (Kaur and Singh, 2002; Kadaverugu et al., 2016). Studies conclude that the land-based treatment and disposal of wastewater is one of the most cost-effective and acceptable methods for wastewater treatment because the soil itself has the capacity to improve the physicochemical and biological properties (Thawale et al., 1999; Vymazal, 2014; Shingare et al., 2017; Panagopoulos, 2021). The studies also conclude that structural properties of soil can be improved to a higher extent and as well as crop productivity when irrigated with treated or untreated wastewater with appropriate management strategies (Angin et al., 2005; Lehrsch et al., 2008). A review by Ungureanu et al. (2020) shows that wastewater has been successfully used for the irrigation of a variety of agricultural crops, such as potatoes, onion, tomatoes, spinach, wheat, maize, rice, etc. A study by Kumari et al. (2015) also concludes that application of distillery wastewater as a liquid fertilizer enhances plant biomass, the number of leaf and pod counts of *Brassica campestris*. The regulatory authorities in India, for instance, the Central Pollution Control Board, also issue guidelines for disposal of wastewater under land-based systems for achieving zero discharge (Central Pollution Control Board, 2019). In the treatment of wastewater containing high TDS, the land disposal treatment system outperforms the other treatment processes. Being cost-effective, easy, and eco-friendly and considering the aesthetic values, land-based treatment systems have attracted the majority of concerned people in recent years, especially in low- and middle-income countries (Ding et al., 2016). A review by Marathe et al. (2021) also concludes that land-based treatment systems are a proven efficient technology for wastewater management with respect to the energy demands, economics, and treatment capacities. Such approaches may be useful to successfully achieve the desired outputs in terms of sustainable wastewater treatment and disposal. However, repetitive research in this area is a mandate to develop high operations skills.

Land-based treatments are employed using plant species having a high salt tolerance capacity. Eucalyptus genus is one such potential plant species, which has been widely employed for different activities such as afforestation, social forestry, wastewater treatment, and carbon sequestration (Yousaf et al., 2021). Numerous studies have been published utilizing Eucalyptus plant species for treatment of a variety of wastewaters, such as paper and pulp mill (Thawale et al., 2015), because of its significant tolerance capacities for saline conditions (Dagar and Minhas, 2016). Nowadays, biosorption has emerged as a new and sustainable technique for wastewater treatment utilizing abundantly available biomaterials, such as agricultural waste (such as rice husk, rice straw, and coconut husk), farmyard manure, etc. Coconut husk has been of great importance and has been extensively used as a bio-adsorbent for the removal of a diverse type of pollutant from water. Coconut-based agricultural wastes have gained wide attention as effective bio-adsorbents due to low cost and significant adsorption potential for the removal of various aquatic pollutants. Abundance, high biosorption capacity, cost-effectiveness, and renewability are the important factors making these materials economical alternatives for water treatment and waste remediation (Bhatnagar et al., 2010). Coconut-based bio-adsorbents show good potential for the removal of various aquatic pollutants. Coconut husk can remove color (Low and Lee, 1990), heavy metals (Sumathi et al., 2005), dyes (Kadirvelu et al., 2000), and phenolic pollutants (Hameed et al., 2008), as well as inorganic anions, such as sulfate (Namasivayam and Sangeetha, 2008), phosphate (Namasivayam and Sangeetha, 2004), thiocyanate (Namasivayam and Sangeetha, 2005), nitrate (Pathak et al., 2006); fluoride (Sathish et al., 2007); atrazine (Sharma et al., 2008), and polycyclic aromatic hydrocarbons (Crisafully et al., 2008) from a variety of wastewaters.

Moreover, Balakrishnan and Rana Rahman (2018) report that rice husk is very effective in the removal of chemical oxygen demand (COD) (76.8%) and BOD (71.6%) in sugar industry wastewater. Other biomaterial (agricultural waste), such as coconut shell, saw dust, rice straw, and cashew nut shell, also removes toxic metal ions and dye from wastewater by ion exchange, surface precipitations, and a complexation mechanism (Abdolali et al., 2014). The organic materials (coconut, rice husk, bagasse, saw dust, rice straw, etc.) have the capability to assimilate/renovate/filter the contaminants (Na, TDS, Cl, color, heavy metals) from wastewater by various physical and chemical processes. It also provides a carbon source for the microorganisms in the rhizosphere that further help in the survival of plants and plant roots in adverse conditions (Thawale et al. 2015). Such organic material not only mitigates salt stress conditions, but also provides additional benefits in terms of plant growth (Maltas et al., 2018; Yuan et al., 2019). Therefore, these organic materials have been selected for the experiment in the present study.

The hypothesis of the study included the treatment strategy of saline wastewater having high TDS (up to 6000 mg/L) as proposed in the Food and Agriculture Organization (FAO) guidelines. According to the FAO, water having TDS up to 2000 mg/L are put under severe restriction use as per the guidelines for water quality for irrigation as the salt accumulates in the crop root zone to a concentration that causes a loss in yield. As a result, it was a mandate for all the industries to treat and discharge the wastewater for irrigation purposes only after meeting the desired criteria defined. Industries mostly release wastewater through reverse osmosis reject having TDS in the range up to 2500–3500 mg/L, which is higher than the prescribed limits and, therefore, face problems in treatment and disposal. Treatment of wastewater to get the desired value using expensive and sophisticated treatment systems was itself a difficult task considering the economic viability. In a similar context, not much attention has been paid globally to the role of natural treatment systems involving forestry plant species to study the impact of saline wastewater irrigation (having higher TDS than prescribed guidelines) other than agricultural crops. Therefore, the present study focused on studying the impact of industrial wastewater with TDS up to 6000 mg/L on forestry species, *Eucalyptus camaldulensis*, and examining the changes in physicochemical and nutrient loads in plants and soil irrigated with saline wastewater.

In the present study, saline wastewater with high TDS (∼6100 mg/L) collected from a common effluent treatment plant (CETP) has been treated using lysimeters aided with low-cost organic raw material, such as rice husk, rice straw, coconut husk, and charcoal, as filter bedding materials (FBM) planted with *Eucalyptus camaldulensis* plant species. In this study, the effect of wastewater on plant growth and the role of FBM in TDS management were continuously monitored. The novelty lies in the treatment of wastewater having very high TDS levels by using a plant combination of FBM, which is a low-cost simple treatment system, and such a system is desirable in developing countries, not only from the point of view of cost, but also in acknowledgment of the difficulty of the reliability of operating potentials of the present complex treatment systems. Most studies (Ali et al., 2012; Thawale et al., 2015; Ganjegunte et al., 2018; Li et al., 2020) report treatment of saline wastewater having low TDS levels. *Eucalyptus camaldulensis*, the forestry tree species, was selected for the present study considering the greater water consumption in comparison with agricultural crops. The reported water use by a eucalyptus tree is about 339–1179 mm/year using sap flow/heat sensors (Khan and Qaiser, 2006). The Eucalyptus species has enormous potential to grow in stress conditions, such as salinity (in the range of 15–25 dS/m) and waterlogged conditions, adaptability to wide ranges of soil types and pH, and high biomass production as well (Shah et al., 2010; Guo et al., 2018). Furthermore, studies incorporating the use of organic raw materials with plants for saline wastewater are also very limited. Therefore, the present study has been designed to determine the effect of different organic raw materials (FBM) on plant growth and TSD management in saline wastewater so as to come up with a cost-effective wastewater treatment technology for various industrial effluents having high TDS.

## Materials and Methods

### Study Site

The study was conducted at CSIR-NEERI, Nagpur, Maharashtra, under field conditions from August 2020 to February 2021. The study area belongs to the agro-ecoregion of Maharashtra, where the climate is characterized by hot summers and mild winters. The mean annual precipitation ranges between 600 and 1000 mm, which covers about 40% of the annual potential evapotranspiration in the range of 1600 to 1800 mm. The moisture availability in the growing period ranges from 90 to 150 days. The common soilscapes in the region are ustorthents, ustropepts, and chromusterts.

### Saline Wastewater Collection and Amendments

Treated effluent was collected from the CETP, Butibori, Nagpur, Maharashtra. The wastewater was amended with a mixture of anhydrous salts, such as potassium chloride (KCl), sodium chloride (NaCl), calcium chloride (CaCl_2_), magnesium chloride (MgCl_2_), and potassium dihydrogen phosphate (KH_2_PO_4_). The salts were dried at 100°C for 2 h and mixed in a homogenizer to get a salt mixture. The salt-amended treated wastewater (moderately saline wastewater) was stored at 4°C as per the standard method prescribed in APHA (2012). The wastewater amendment was done to achieve the desired TDS levels in the range up to 6000 mg/L.

### Experimental Setup

**FIGURE 1:**
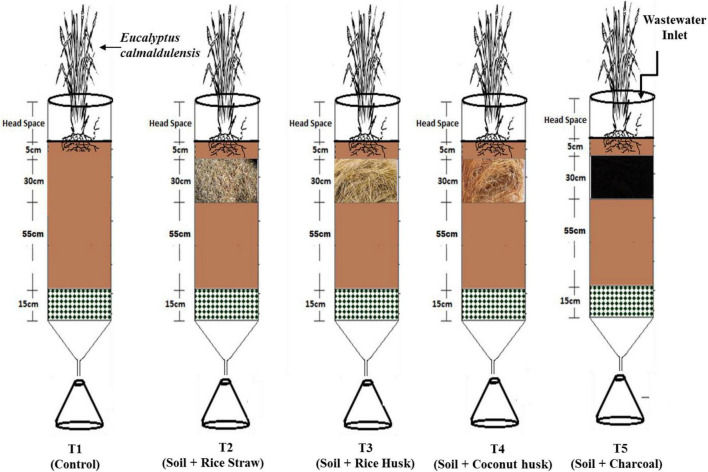
Experimental setup of different lysimeter treatments.

The experimental setup included five treatments (four plus one control) as shown in Figure 1 and Table 1 to elucidate the role of plant-derived FBM and phyto-treatment capability of *Eucalyptus camaldulensis* for management of moderately saline wastewater having high TDS. Acrylic columns of 20 cm diameter and 120 cm height with a port at the bottom to collect the leachate were used in the present study. All the columns were filled with a mixture of sand (<2 mm), marble chips (10–15 mm), black cotton soil, and plant-derived FBMs, which included coconut husk, rice husk, rice straw, and charcoal.

**TABLE 1:** Treatment details of experiment and the FBM.

S. No	Lysimeter number	Depth of bedding material (cm)	Treatment details
1	T1 (Control)	−	Soil + *Eucalyptus camaldulensis* without filter bedding material
2	T2	30 cm	Soil + *Eucalyptus camaldulensis* + rice straw
3	T3	30 cm	Soil + *Eucalyptus camaldulensis* + rice husk
4	T4	30 cm	Soil + *Eucalyptus camaldulensis* + coconut husk
5	T5	30 cm	Soil + *Eucalyptus camaldulensis* + activated charcoal

Gravel derived from the basaltic rock fragments was crushed up to the size of 15–100 mm and was used as the bottommost layer in the columns to mimic the parent rock layer. Above this, small-sized gravel (10–15 mm) was placed, followed by sand. All lysimeters were rinsed with distilled water and filled with a layer of a mixture of sand (10–15 mm) and marble chips (<2 mm). Black cotton soil (up to 55 cm) filled each of the columns above the sand/marble chip layer. A layer of different FBMs (30 cm) followed by a topsoil layer (10 cm) was made in each of the lysimeters. A salt-tolerant plant species, *Eucalyptus camaldulensis*, was planted in each lysimeter (Figure 1) and allowed to adapt to treatment conditions initially for 15 days irrigating them with tap water.

The five treatments designed in this study were as follows; control without FBM + *Eucalyptus camaldulensis* + soil (T1); soil + rice straw + *Eucalyptus camaldulensis* (T2); soil + rice husk+ *Eucalyptus camaldulensis* (T3); soil+ coconut husk+ *Eucalyptus camaldulensis* (T4), and soil + charcoal + *Eucalyptus camaldulensis* (T5). Each treatment has triplicate replications with a constant depth of 30 cm for FBM. Results obtained for physical characteristics of FBM are shown in Table 2.

**TABLE 2:** Physical characteristics of FBM used in the study.

S. No.	Treatments	Bedding material	Parameters		
			Bulk Density (g/cm^3^)	Maximum water holding capacity (%)	Porosity (%)
1	T1 (Control)	Soil	1.43 ± 0.01	55.78 ± 0.06	64.33 ± 0.35
2	T2	Rice straw	0.13 ± 0.02	197.21 ± 0.045	19.41 ± 0.23
3	T3	Rice husk	0.09 ± 0.01	217.07 ± 0.03	45.52 ± 0.03
4	T4	Coconut Husk	0.20 ± 0.01	446.68 ± 2.01	92.53 ± 0.21
5	T5	Charcoal	0.37 ± 0.03	100.32 ± 1.23	40.32 ± 0.05

*− Values after (±) represents standard deviation (SD).*

### Physicochemical Analysis of Moderately Saline Wastewater and the Percolate

Different physicochemical characteristics, such as color, electrical conductivity (EC), pH, total hardness, TDS, BOD, COD, alkalinity, sulfate, sodium, potassium, calcium, magnesium, nitrates, ammonia, and heavy metals were analyzed according to standard protocol (APHA, 2012). pH and EC of wastewater was determined by a pH and EC meter (Hach Model – VSI = 06) as described by Jackson (1973). TDS analysis was performed using the gravimetric method. A modified Winkler-azide method was adopted for BOD analysis using a BOD incubator. Kjeldahl distillation was performed for estimation of ammonia in wastewater. The total hardness, calcium, and magnesium were determined by the EDTA titrimetric method. Similarly, phosphates, sulfates, and nitrates were determined by using the spectroscopic method. Sodium and potassium concentrations were determined by using a flame photometer. The heavy metal analysis (Zn, Cu, Fe, Mn, Pb, Cr, and Co) was performed using inductively coupled plasma–optical emission spectrometry (ICP-OES) (ISO, 2001). The method incorporates acid digestion using nitric acid for determination of total heavy metals in wastewater. The closed reflux method followed by spectrophotometric analysis was performed for COD analysis (Li et al., 2009).

### Physicochemical Characterization of Soil

Soil physicochemical characteristics were determined at the initial and final stages of the experiment. Soil samples from each of the lysimeters were collected, air dried, and sieved through a 2-mm sieve. Soil pH and EC were determined in the suspension of the soil in water by using a water and soil analysis kit (Hach Model – VSI = 06) as described by Jackson. (1973). Na^+^, K^+^, Ca^2+^, and Mg^2+^ ion concentrations were determined by flame photometry, and the heavy metals were analyzed by ICP-OES (ISO, 2001). The metals in the soil sample were extracted by acid digestion using nitric acid; 0.5 g of soil sample was taken in a Teflon vessel, and 10 ml of nitric acid was taken in a Teflon vessel and digested for 2 h in microwave digester, diluted with distilled water, and filtered, and volume make up was done with distilled water in a 100-ml volumetric flask and later stored at 4°C for ICP-OES analysis. Bulk density, porosity, and maximum water-holding capacity were determined by using the KR box method (Keen and Raczkowski, 1921). Organic carbon and total nitrogen were estimated by the Walkley and Black method (Walkley and Black, 1934) and the modified Kjeldahl method (Bremner and Mulvaney, 1983). Total phosphorous of soil samples was determined according to the Olsen method (Richards, 1954; Olsen and Sommers, 1982).

### Plantation and Analysis of Plant Growth Parameters

*Eucalyptus camaldulensis* of height 30 and 8 cm root length were planted in the lysimeters. During the period of acclimatization, each lysimeter was initially fed with tap water for 15 days. After that, moderately saline wastewater was fed to each lysimeter at a rate of 200 m^3^ day^–1^ ha^–1^ (i.e., 312 ml per 0.0156 m^2^ area of lysimeter). The hydraulic loading rate can be calculated using the following equation (Thawale et al., 2006).


$$\mathrm{Hydraulic loading rate}\left(m^{3}day^{-1}\right)=\mathrm{Total}\mathrm{flow}\mathrm{applied}$$

Each *Eucalyptus camaldulensis* plant in each treatment was monitored both before and after treatment for growth parameters, such as height (cm), root length, number of shoots, fresh and dry weight, chlorophyll content, total Kjeldahl nitrogen, heavy metals, and uptake of salts in terms of calcium, magnesium, sodium, potassium, and phosphorus, respectively. Height measurements were taken manually. Dry weight was determined by oven drying at 70°C for 48 h (Pereira et al., 2020). To determine the salt uptake by *Eucalyptus camaldulensis*, leaves, stems, and roots were cut into small pieces and dried to a temperature of 70°C for 48 h. Dried samples were crushed into fine powder with a 0.5-mm sieve and stored in zip-locked bags for further analysis. Concentration of total nitrogen was determined by using a modified micro-Kjeldahl digestion procedure (Bremner and Mulvaney, 1983). Total P, K, Na, Ca, Mg, Cd, Mn, Fe, Cu, Pb, and Zn were analyzed by a dry ash digestion method: 1 g of plant sample was taken into crucible and was put into a muffle furnace at the temperature of 550°C for 8 h (Campbell and Plank, 1998; Miller, 1998). The ash was collected and dissolved in 3M hydrochloric acid (HCl) (10,ml) for the estimation of heavy metals and phosphorous, respectively (Horneck and Miller, 1998). Later, the concentration of P, K, Na, Ca, Mg, Cd, Mn, Fe, Cu, Pb, and Zn were determined by using ICP-OES, whereas concentration of P was determined by using the vanadate/molybdate method (Chapman and Pratt, 1961). Chlorophyll content analysis was performed before harvest by using a spectroscopic method with 1 g of fresh homogenous plant leaf material. The homogenized leaf material was mixed with 20 ml of 80% acetone, and 0.5 g of MgCO_3_ was added. The sample was kept in a refrigerator for 4 h at 40°C. Later, the sample was centrifuged at 500 rpm for 5 min, and the supernatant was transferred into a 100-ml volumetric flask, and volume make up was made with 80% acetone. The absorbance was taken at two wavelengths, 645 and 663 nm, respectively, with 80% acetone as a blank (Kamble et al., 2015).

### Statistical Analysis

The data was analyzed statistically using one-way ANOVA followed by Tukey’s honestly significant difference (HSD) test. The test was performed for the parameters of soil and wastewater quality before and after treatment to assess the efficiency of FBM in salt removal in between the different treatments and the treatment efficiency and the associated plant growth. The significant differences were tested at 95% confidence level (*p* < 0.05).

## Results and Discussion

### Initial Characterization of Saline Wastewater

The various physicochemical characteristics of the effluent analyzed in this study are shown in Table 3. As per the results, wastewater possessed high TDS (6143.33 ± 5.77 mg/L) and EC (11.21 ± 0.74 dS/m). The COD and BOD were in the range of 142.42 ± 10.31 and 12 ± 2.0 mg/L, respectively. The concentration of sodium (Na^+^) ions and bicarbonates (HCO_3_^–^) were found to be 1170 ± 1.04 and 433.43 ± 4.62 mg/L, respectively. Concentration of the potassium (K^+^) divalent cations, calcium (Ca^2+^), magnesium (Mg^2+^), and SAR was found to be 95.14 ± 2.41, 2305.74 ± 15.51, and 1237.12 ± 10.24 mg/L and 2.21, respectively. Heavy metal concentrations were negligible. The characterization of amended wastewater implied that it belonged to the “severe restriction for use” category in irrigation as it had higher values of parameters such as EC, TDS, and SAR (Kandiah, 1987).

**TABLE 3:** Initial physicochemical characterization of moderately saline wastewater.

S. No.	Parameters	Wastewater	Indian standards
1	pH	8.55 ± 0.02	5.5–9.0
2	EC (dSm−1)	11.21 ± 0.74	−
3	TDS (mg/L)	6143.33 ± 5.77	2,100
4	Total Hardness (mg/L)	3542.86 ± 10.66	−
5	Calcium (mg/L)	2305.74 ± 15.51	−
6	Magnesium (mg/L)	1237.12 ± 10.24	−
7	COD (mg/L)	142.42 ± 10.31	−
8	BOD (mg/L)	12 ± 2	100
9	Bicarbonates (mg/L)	433.43 ± 4.62	−
10	Phosphate (mg/L)	10 ± 1.73	−
11	Sulfate (mg/L)	101.12 ± 1.89	1000
12	Nitrate (mg/L)	14.33 ± 1.15	−
13	Ammonia (mg/L)	9.57 ± 2.75	−
14	Sodium (mg/L)	1170 ± 1.04	−
15	Potassium (mg/L)	95.14 ± 2.41	−
16	Sodium adsorption ratio	2.21 ± 0	<26
17	Cadmium (Cd) (mg/L)	ND	0.05
18	Chromium (Cr) (mg/L)	0.03 ± 0.01	2
19	Copper (Cu) (mg/L)	0.02 ± 0.01	3
21	Iron (Fe) (mg/L)	0.2 ± 0.03	3
22	Manganese (Mn) (mg/L)	0.04 ± 0.01	2
23	Nickel (Ni) (mg/L)	0.03 ± 0	3
24	Lead (Pb) (mg/L)	ND	0.1
25	Zinc (Zn) (mg/L)	0.07 ± 0.01	15

*− Values after (±) represents standard deviation (SD). ND, not detected.*

### Initial Characterization of Soil

The soil used in the study had a clayey texture, and the content of sand, silt, and clay in the soil were 33–35.9, 12.1–15.7, and 49.7–54.4%, respectively. pH, EC, and organic carbon were found to be 7.66 ± 0.03, 0.15 ± 0.01 dSm^–1^, and 0.8 ± 0%, respectively (Table 4). Cation exchange capacity (CEC) and exchangeable sodium percentage (ESP) of the soil were 34.99 ± 0.85 C^+^ mol/kg and 1.37 ± 0.14%, respectively. The soil fell into the moderately alkaline category based on analytical results. Soils with moderately alkaline pH support significant plant growth (Lauchli and Grattan, 2012). In addition, the ESP of the soil did not exceed the limit defined for sodic soil (>15) and, therefore, did not have any adverse impact on soil and plants (Amacher et al., 2007).

**TABLE 4:** Initial physicochemical characterization of soil.

S. No	Parameters	Soil
**Physical properties**		
1	Bulk density (g/cm^3^)	1.43 ± 0.01
2	Maximum water holding capacity (%)	55.78 ± 0.06
3	Porosity (%)	64.33 ± 0.35
4	Sand (%)	33–35.9
5	Silt (%)	12.1–15.7
6	Clay (%)	49.7–54.4
7	Textural class	Clay
**Chemical properties**		
1	pH	7.66 ± 0.03
2	EC (dS/m)	0.15 ± 0.01
3	Organic carbon (%)	0.8 ± 0
4	Organic matter (%)	0.15 ± 0.01
**Exchangeable cations**		
1	Sodium (Cmol^+^/kg)	0.19 ± 0.01
2	Potassium (Cmol^+^/kg)	0.99 ± 0.06
3	Calcium (Cmol^+^/kg)	4.31 ± 0.01
4	Magnesium (Cmol^+^/kg)	6.36 ± 0.02
5	Cation Exchange Capacity (Cmol^+^/kg)	34.99 ± 0.85
6	Exchangeable Sodium Percent (%)	1.37 ± 0.14
**Macro nutrients (%)**		
1	Nitrogen	0.19 ± 0.01
2	Phosphorous	0.04 ± 0.01
3	Potassium	0.37 ± 0.01

*− Values after (±) represents standard deviation (SD).*

### Assessment of Leachate Quality

**FIGURE 2:**
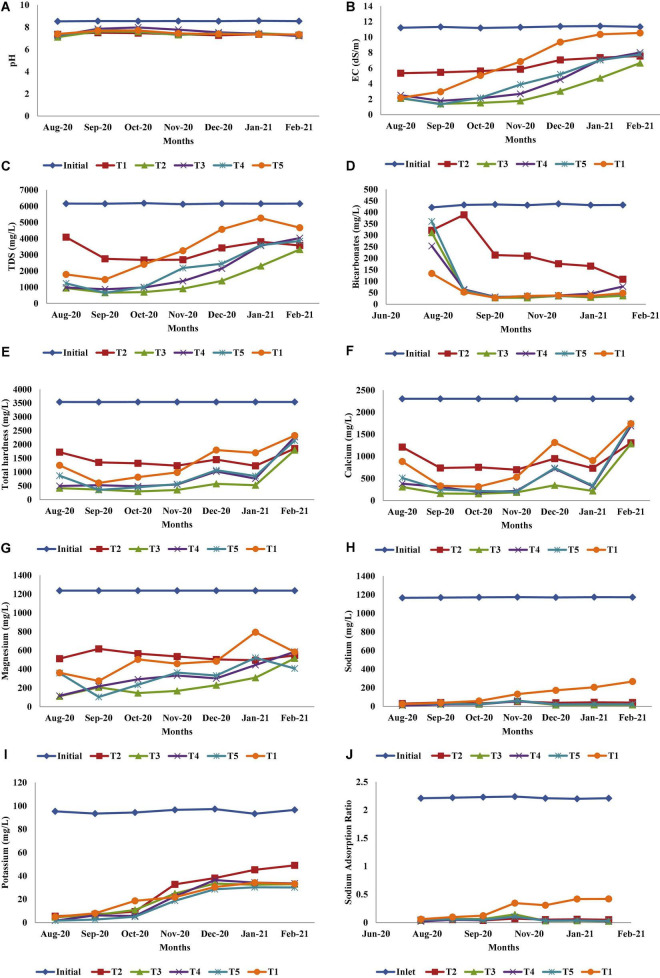
**(A–J)** Variation in different physicochemical characteristics of leachate in different treatments throughout the study with respect to the pH **(A)**, EC **(B)**, TDS **(C)**, bicarbonates **(D)**, total hardness **(E)**, calcium **(F)**, magnesium **(G)**, sodium **(H)**, potassium **(I)**, and SAR **(J)**.

**FIGURE 3:**
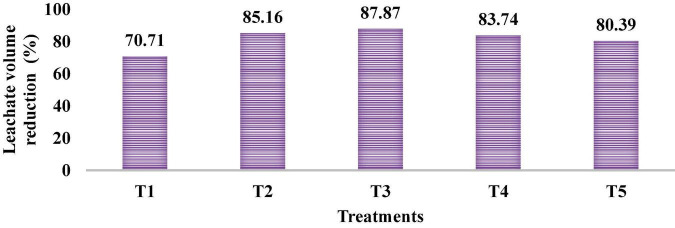
Overall percentage reduction in the leachate at the end of the experiment.

The physicochemical parameters of the leachate are given in Table 5. The average TDS concentration in the leachates in different treatments at the end of the experiment were T1: 3348.2 ± 84.4 mg/L, T2: 3288.8 ± 64.2 mg/L, T3: 1461.7 ± 70.2 mg/L, T4: 1992.3 ± 104.8 mg/L, and T5: 2138.8 ± 100.1 mg/L. Therefore, the order of the salt accumulation in the respective treatments was T3 > T4 > T5 > T2 > T1. Significant differences were also found among different treatments for the parameters, namely, EC, TDS, Na, and SAR (*p*-value > 0.05). Heavy metals were not detected in the leachate because the initial concentrations were very low. The changes in various physicochemical properties of leachate during the period of study are shown in Figures 2A–J. Significant removal of heavy metals from the leachate were also found in the present study because of adsorption on FBM or organic raw materials consisting of coconut husk, rice husk, straw, and charcoal. Likewise, coconut coir powder used for wastewater treatment also showed effective removal of heavy metals, such as copper, nickel, and cadmium (Aravind et al., 2017). Percentage leachate volume reduction at the end of the experiment is also shown in Figure 3. The reduction in the volume of the leachate is due to the application of FBM in the lysimeters, which acted as the volume storage compartments. Literature is also available that shows FBMs (specifically coconut and rice husk) have high porosity and water-holding capacity; as a result, it accumulates high volumes of water and thereby the nutrients within itself (Ahiduzzaman and Islam, 2015; Ravindranath and Radhakrishnan, 2016).

**TABLE 5:** Average values of physicochemical characteristics of the leachates throughout the study.

S. No.	Parameters	Average physicochemical characteristics of leachate throughout the study					
	Treatments	T1	T2	T3	T4	T5	*p*-value
1	pH	7.37 ± 0.07^a^	7.4 ± 0.06^a^	7.57 ± 0.06^b^	7.47 ± 0.09^ab^	7.47 ± 0.07^ab^	0.015
2	EC (dSm^–1^)	6.76 ± 0.42^a^	6.33 ± 0.18^a^	3.03 ± 0.25^b^	4.1 ± 0.32^b^	4.22 ± 0.5^b^	0.001
3	TDS (mg/L)	3348.16 ± 84.41^a^	3288.83 ± 64.22^a^	1461.74 ± 70.15^b^	1992.3 ± 104.75^b^	2138.8 ± 100.05^b^	0.001
4	Total Hardness (mg/L)	1354.41 ± 117.74^a^	1451.55 ± 38.92^b^	618.74 ± 98.81^b^	873.57 ± 148.84^ab^	900.83 ± 121.88^ab^	0.002
5	Calcium hardness (mg/L)	860.03 ± 82.11^ab^	912.35 ± 44.58^a^	378.15 ± 46.28^c^	546.72 ± 87.25^bc^	569.48 ± 77.53^bc^	0.001
6	Magnesium hardness (mg/L)	494.38 ± 50.33^a^	539.2 ± 43.51^a^	240.59 ± 33.84^b^	326.85 ± 35.8^b^	331.35 ± 32.08^b^	0.001
7	COD (mg/L)	ND	ND	ND	ND	ND	−
8	BOD (mg/L)	ND	ND	ND	ND	ND	−
9	Nitrite mg/L	ND	ND	ND	ND	ND	−
10	Bicarbonates (mg/L)	53.26 ± 2.55^b^	226.53 ± 7.16^a^	75.31 ± 2.81^b^	77.84 ± 4.13^b^	86.4 ± 3.09^b^	0.001
11	Phosphate (mg/L)	ND	ND	ND	ND	ND	−
12	Sulfate (mg/L)	ND	ND	ND	ND	ND	−
11	Nitrate (mg/L)	ND	ND	ND	ND	ND	−
13	Ammonia (mg/L)	ND	ND	ND	ND	ND	−
14	Sodium (mg/L)	128.08 ± 22.56^a^	39.55 ± 1.55^b^	24.31 ± 5.62^b^	24.99 ± 5.98^b^	29.79 ± 8.24^b^	0.001
15	Potassium (mg/L)	21.56 ± 2.24^a^	26.7 ± 4.51^a^	20.78 ± 3.51^a^	20 ± 3.74^a^	16.66 ± 4.79^a^	0.529
16	SAR	0.25^a^	0.05^b^	0.06^b^	0.04^b^	0.05^b^	0.0001

*Values after (±) represents standard deviation (SD). The means with different letter differ are significantly different at p < 0.05 (Tukey’s HSD test). ND, Not Detected.*

### Effect of Moderately Saline Wastewater on Soil Physicochemical Properties

The physicochemical properties of the soil after treatment are shown in Table 6. The bulk density decreased, whereas maximum water-holding capacity and porosity increased after the treatment. The values of bulk density of the soil were observed to be 1.43 g cm^–3^, but after the treatments, it was found to be in the range of 1.21–1.36 g cm^–3^. The reduction of bulk density was observed to be in the range of 3.50–15.38%. Similarly, maximum water-holding capacity and porosity increased in soil after treatments as compared with controls and were in the range of 56.29–63.29 and 65.25–68.07%, respectively. Similar trends were also reported by Biswas et al. (2017). The pH was found to be close to neutral values initially; however, after treatments there was slight increase in pH among the different treatments due to salt accumulation. The oxidation of organic matter by microbial activity and increase in the nutrients can be responsible for the increase in the pH of soil irrigated with saline effluent (Vadivel et al., 2019). The organic carbon and organic matter content were significantly increased in all the treatments as compared with initial values, and the range was 0.96–1.37 and 1.68–2.33%. Bulk density has a negative correlation with the organic matter and carbon as well as soil aggregation (Biswas and Mojid, 2018). A similar decrease in bulk density with the increase of organic carbon and matter is also reported in a previous study (Novara et al., 2019). Similarly, nutrient status (N, P, and K) differed significantly among the treatments as compared with initial values. The highest increase in concentration of NPK was observed in treatments T3 and T4 in topsoil, corresponding to the high nutrient-holding capacity of coconut and rice husk; however, a decreased concentration of nitrogen was observed in T1, which may be due to leaching of the nutrient along with the leachate (Lakshmi et al., 2019).

**TABLE 6:** Changes in physicochemical properties of soil after the application of wastewater.

Treatment	Initial Soil	Soil After treatment					*p*-value
		T1	T2	T3	T4	T5	
**Physical parameters**							
Bulk density (g/cm^3^)	1.43 ± 0.01	1.28 ± 0.01^d^	1.21 ± 0^g^	1.36 ± 0^b^	1.32 ± 0.01^c^	1.29 ± 0.01^d^	0.0001
Maximum Water Holding Capacity (%)	55.78 ± 0.06	57.05 ± 0.45^g^	62.15 ± 0.02^b^	63.29 ± 0.19^a^	62.13 ± 0.15^b^	56.29 ± 0.3^f^	0.0001
Porosity (%)	64.33 ± 0.35	65.76 ± 0.4^c^	67.27 ± 0.15^b^	68.05 ± 0.29^a^	66.07 ± 0.07^c^	66.29 ± 0.26^c^	0.0001
Textural class	Clay	Clay	Clay	Clay	Clay	Clay	
**Chemical properties**							
pH	7.66 ± 0.03	7.63 ± 0.08^c^	7.66 ± 0.04^c^	8.59 ± 0.07^a^	8.57 ± 0.07^a^	7.68 ± 0.01^c^	0.001
EC (dS/m)	0.15 ± 0.01	1.07 ± 0.015^bc^	0.65 ± 0.02^c^	0.34 ± 0.019 ^e^	0.36 ± 0.02 ^e^	0.55 ± 0.02 ^d^	0.0001
Organic Carbon (%)	0.8 ± 0	0.96 ± 0.05^ab^	1.34 ± 0.05^ab^	1.25 ± 0.05^b^	1.37 ± 0.05^ab^	1.37 ± 0.05^ab^	0.034
Organic Matter (%)	1.40 ± 0.01	1.63 ± 0.09^ab^	2.28 ± 0.09^ab^	2.13 ± 0.09^b^	2.33 ± 0.09^ab^	2.33 ± 0.09^ab^	0.034
**Total nutrients (%)**							
Total Nitrogen	0.19 ± 0.01	0.17 ± 0^e^	0.2 ± 0^d^	0.21 ± 0^cd^	0.22 ± 0.02^cd^	0.2 ± 0^d^	0.0001
Total Phosphorus	0.04 ± 0.01	0.05 ± 0.007^b^	0.08 ± 0.007^ab^	0.08 ± 0.007^ab^	0.07 ± 0.012^ab^	0.08 ± 0.008^a^	0.002
Potassium	0.37 ± 0.01	0.39 ± 0.02^d^	0.43 ± 0.02^cd^	0.44 ± 0.02^bcd^	0.49 ± 0.02^ab^	0.46 ± 0.03^abc^	0.0001
**Exchangeable cations**							
Ca (Cmol^+^/kg)	4.31 ± 0.01	11.88 ± 1.3^a^	7.42 ± 1.2^a^	6.59 ± 0.64^a^	6.08 ± 0.64^a^	8.44 ± 0.78^a^	0.022
Mg (Cmol^+^/kg)	6.36 ± 0.02	9.66 ± 1.3^a^	7.19 ± 0.78^a^	7.02 ± 1.1^a^	6.81 ± 1.1^a^	9.03 ± 1.34^a^	0.520
Na (Cmol^+^/kg)	0.19 ± 0.01	8.73 ± 0.27^e^	4.53 ± 0.13^b^	2 ± 0.04^b^	3.68 ± 0.09^cd^	3.97 ± 0.06^de^	0.0001
K (cmol^+^/kg)	0.99 ± 0.06	0.77 ± 0.03^c^	0.01 ± 0^b^	0.31 ± 0.0^d^	0.16 ± 0.04^c^	0.22 ± 0.01^c^	0.0001
CEC (Cmol^+^/kg)	34.99 ± 0.85	35.3 ± 0.77^bcd^	33.61 ± 3.1^ab^	40.11 ± 0.49^cd^	41.8 ± 1.45^a^	38.14 ± 1.12^cd^	0.0001
ESP (%)	1.37 ± 0.14	24.73 ± 0.75^e^	13.47 ± 0.42^d^	4.98 ± 0.63^cd^	8.80 ± 0.15^e^	10.4 ± 0.16^e^	0.0001

*Values after (±) represents standard deviation (SD). The means with different letter differ significantly at p < 0.05 (Tukey’s HSD test).*

The initial values of exchangeable cations of soil Na^+^, K^+^, Ca^2+^, and Mg^2+^ were observed to be 0.19, 0.99, 4.31, and 6.36 Cmol^+^/kg, respectively. It was observed that there was a decrease in the concentration of the exchangeable cations in soil after the application of saline wastewater as shown in Table 6 because of the adsorption of cations on the FBM. EC, CEC, and ESP were also decreased in the soil after treatment; however, increased values were found for the control (T1) treatment as compared with treatments containing FBM, which may be attributed to the direct loading of a high concentration of salts from saline wastewater to the soil due to the absence of FBM (Kumar and Chopra, 2010, 2011). In addition, the percentage increase for EC, CEC, and ESP was lower in soil after treatment containing FBM, clearly establishing the significance of FBM in the management of salinity through bio-adsorption. Similar results were observed in other studies showing the adsorption of TDS using rice and coconut husk as bio-adsorbents (Che bt Man et al., 2015; Mor et al., 2016; Aljeboree et al., 2017).

Though the EC (dScm^–1^) in all treatments after wastewater application increased, the saline conditions were not developed. EC in the present study was in the range of 0–2 dSm^–1^ and show no impact of saline wastewater to the soil and, therefore, is classified as nonsaline (Prasad and Maurya, 2014). The ESP values among all treatments were also below 15%, showing that sodic conditions were not developed in any of the treatments except for controls (Amacher et al., 2007). On comparing the characteristics of initial soil and soil after treatment, a significant difference was observed at *p*-value < 0.05 soil considering the parameters such as bulk density, water-holding capacity, and porosity as well as exchangeable Na, K, Ca, CEC, and ESP. Significance differences among the treatments and control confirmed the better treatment capacity of FBMs, and the order of the treatment capacity was found to be (T3) > (T4) > (T5) > (T2) > Control (T1).

### Role of Filter Bedding Materials

The organic materials can directly be used as adsorbents for the treatment of wastewater containing various pollutants because of their higher availability and lower costs. Similarly, agricultural waste such as coconut husk, sawdust, rice husk, and other waste, which are widely available in nature can be used to remove unwanted toxic pollutants from wastewater, including inorganic salts, dyes, oils, and heavy metals (Nduka, 2012). The bedding material acts as adsorbent for management of organic and inorganic ions. Many researchers use low-cost organic raw materials, such as rice husk, rice straw, coconut husk, sawdust, peanut shells, and charcoal for wastewater treatment for effective removal of TDS, COD, BOD, color, and dissolved solids from various industrial effluents (Yadav et al., 2010; Aljeboree et al., 2017; Yousaf et al., 2021). The removal of basic dye from wastewater has also been investigated using natural adsorbents with higher removal rates (Che bt Man et al., 2015). In the present study, more promising results were obtained in terms of TDS removal and removal of other pollutants from wastewater, such as COD, BOD, and heavy metals.

The total inlet TDS applied to each of the lysimeters was 406.34 g for a period of 7 months. TDS or salt retention in the control treatment (T1) was the least, and therefore, there was the highest leaching of salts in percolate, which was collected from the control treatment. The other treatments, which had FBM, showed significant salt reduction and thereby lower TDS levels in the percolate (leachate) as shown in Table 7. Treatments T3 and T4 showed the best results as compared with T5, T2, and T1. The observed trend of efficiency (average) for salt removal in terms of TDS among different treatments was T3 (76.21%) > T4 (67.57%) > T5 (65.18%) > T2 (46.46%) > T1 (45.5%). In addition, the highest TDS accumulation was in T3 followed by T4, thereby with a lesser concentration in leachate. High TDS removal can be directly correlated with the presence of rice and coconut husk as both of them are reported to have high accumulations of salts and have a longer shelf life in soil, making them the best suitable FBM (Ahiduzzaman and Islam, 2015; Ravindranath and Radhakrishnan, 2016). Charcoal also shows promising results for heavy metal and salt removal from industrial wastewaters as in the present study (Agarwal and Singh, 2017). Similarly, numerous studies show successful utilization of rice husk for pollutant removal (Srivastava et al., 2008; Akhtar et al., 2010; Singh and Singh, 2012; Hegazi, 2013; Singh et al., 2020). Apart from that, coconut and rice husk show higher values of maximum water-holding capacity as compared with others (Table 1). Therefore, it can be concluded that the large surface area and water-holding capacity facilitate the higher adsorption of inorganics from the effluents in comparison with other FBMs and, therefore, show exemplary performance (Hariz et al., 2015).

**TABLE 7:** Overall salt accumulation in terms of TDS (g) in different treatments throughout the study.

Treatments	TDS (g) Salt accumulation in lysimeters	TDS in percolate (g)	Removal efficiency (%)
**Total inlet TDS to each of the treatment is 406.34 g**			
Treatment (T1)	184.88	221.46	45.5%
Treatment (T2)	188.79	217.55	46.46%
Treatment (T3)	309.67	96.67	76.21%
Treatment (T4)	274.56	131.78	67.57%
Treatment (T5)	264.85	141.49	65.18%

Various mechanisms are involved in the adsorption of various pollutants using natural adsorbents. Coconut husk and coconut coir consist of hollow internal conduits, xylem and phloem, where xylem facilitates the uptake of water and phloem serves the purpose of transport of dissolved solids into the plant vacuoles, pits, voids, and lacunae or lumina (Kato et al., 1997). Similarly, the surface affinity and adsorbent properties of rice husk play important roles in TDS removal from wastewater. The percentage removal of pollutants depends upon the pH of the wastewater: At lower pH, the pollutant sorption capacity of the rice husk is less, and at higher pH, the pollution sorption capacity increases (Pathak et al., 2016).

The larger specific surface area and higher water-holding capacity in coconut husk, rice husk, charcoal, and rice straw facilitate a higher percentage absorption of inorganic salts and other pollutants from the wastewater. Hence, the adsorption capacity of the pollutants is determined by the surface area and porous structure of the adsorbents along the chemical interactions between the functional groups of adsorbents and inorganic ions present in the wastewater. Therefore, the higher removal of TDS in this study may be due to the adsorption–desorption mechanism due to physical interactions at a dynamic equilibrium (Karaca et al., 2005). The variation in the removal capacity of different adsorbents may also be due to inhomogeneity in shapes, surface areas, particle shapes, surface area, morphology, pore size of the adsorbents, and retention of targeted pollutants on the adsorbents because the adsorption/desorption is nonstoichiometry process (Gallego Piñol et al., 2009). Similar mechanisms are also reported for the treatment of brewery and beverage wastewater using rice husk as adsorbent (Nduka et al., 2007). Similar results are also shown in the study of Matisová and Škrabáková (1995). In addition to this, Van der Waals and London forces also play major roles in the physical interactive removal of heavy metals and other pollutants from wastewater (Nduka, 2012).

Significant variations were observed in pH and other parameters among all treatments. Consecutively, negligible concentrations of phosphate, sulfate, nitrate, ammonia, BOD, and COD were observed in the treatments containing rice and coconut husk leachate, making the systems’ efficiency range between 65 and 76% and justifies the role of FBM in management of TDS as compared with control and other treatments. FBMs such as rice straw and charcoal also show significant results but had lower efficiencies as compared with rice and coconut husk.

In the context of degradability of the FBM, coconut husk, rice husk, and rice straw contain high lignocellulose biomass, which consists of a complex structure of cellulose, hemicellulose, and lignin. These complex compounds are not easily degradable and, therefore, take longer for degradation by microorganisms (Kumar et al., 2020). Similarly, the high silica content of coconut husk, rice husk, and rice straw also slows down the growth of microorganisms, ultimately slowing down their degradation rate (Anuar et al., 2018; Motlagh et al., 2020). The high silica content in rice husk, rice straw, and coconut husk are also responsible for antioxidative properties inhibiting the oxidation process (Kim et al., 2012). Also, lignocellulosic biomass and its structural stability has high tendency to adhere the cations, such as heavy metals and salts, facilitating effective adsorption (Kumar and Chandra, 2020). Similarly, higher adsorption of salts was obtained in the present study in different treatments. We further note that, as the FBM was kept in between the two soil layers, therefore, the continued application of wastewater to the system may be responsible for depletion of oxygen in the lysimeters and thereby slower degradation of the FBM. Furthermore, FBM or bio-adsorbents in general have certain adsorption capacity for salts; therefore, it can be concluded here that the gradual increase in the nutrient contents in the leachate in different treatments toward the end of the experiment could be because of the threshold capacity for each of FBM adsorption.

### Assessment of Plant Growth Fed With Wastewater

*Eucalyptus camaldulensis* showed good response to wastewater treatment having high TDS in terms of growth, chlorophyll content, root/shoot weight, and uptake of sodium and potassium and other nutrients. In addition, the increase in plant height and significant nutrient uptake showed the salt-tolerance capacity of *Eucalyptus camaldulensis*. Changes in the growth parameter of the plant during the period of study are shown in Table 8. The average percentage increase in the plant height among all treatments was found to be 118.4%. A similar study by other researchers showed significantly increased height of plants treated with wastewater (Thawale et al., 1999; Ledesma et al., 2016). Nutrient content was analyzed in leaves, roots, and shoots of the plant, and the results show that uptake of sodium, potassium, calcium, magnesium, total nitrogen, and total phosphorus concentrations were higher in treatments as compared with the control (T1). Similar enhanced nutrient uptake is also reported in other studies (Edelstein et al., 2009; Liu et al., 2016). However, the uptake was very low as compared to the present study. Therefore, the present study more readily favors the better TDS management because of the significant nutrient or salt uptake by plants. Similar conclusions are also made by other researchers that phytoremediation could be a better approach for the management and disposal of saline wastewater with higher TDS (Ghotbizadeh and Sepaskhah, 2015; Roongtanakiat and Akharawutchayanon, 2017; Golabi et al., 2018; Yousaf et al., 2021). Comparison of means between initial and final values of root and shoot weight, plant height, nutrient uptake, and chlorophyll content are shown in Tables 8, 9. The highest values were found in treatments T3 and T4 followed by T2 and T5 and the least in the control (T1). However, the nutrient uptake was more or less similar in all treatments except for T1. Plant shoots have more distributed nutrients in all treatments as compared with roots. Mane et al. (2011) show increased root and shoot length in the presence of saline conditions, which can be positively correlated with the findings of our study. The findings of Keshtkar et al. (2016) were also similar to the findings of the present study.

**TABLE 8:** Assessment of growth parameters of *Eucalyptus camaldulensis* after wastewater application.

Treatment	Water content (%)	Height (cm)	No of new shoots (cm)	Length of roots (cm)	Dry weight Leaf (gm)	Dry weight Shoot (gm)	Dry weight root (gm)	Chlorophyll A (μg/g)	Final Chlorophyll B (μg/g)	Total chlorophyll (μg/g)	Final Chlorophyll A/B
Initial	8 ± 0.02^d^	30.12 ± 1.76^c^	1 ± 0^f^	8 ± 0.86^d^	−	−	−	0.35 ± 0.0^d^	0.11 ± 0.02^c^	0.46 ± 0.1^d^	0.33 ± 0.07^e^
**After treatment**											
T1	11.65 ± 0.15^c^	80.56 ± 0.03^c^	4 ± 0^d^	27.31 ± 0.81^c^	3.2 ± 1.2^d^	37.1 ± 0.7^c^	45.6 ± 0.85^a^	6.8 ± 0.1^c^	4.2 ± 0.4^a^	11 ± 0.32^c^	1.62 ± 0.4^d^
T2	10.64 ± 1.29^c^	89.65 ± 0.03^b^	3 ± 0^e^	29.26 ± 0.02^c^	4.5 ± 1.3^c^	38.2 ± 0.6^ac^	46.9 ± 0.24^a^	7.2 ± 0.2^b^	4.3 ± 0.29^a^	11.5 ± 0.24^a^	1.67 ± 0.31^c^
T3	12.18 ± 0.14^bc^	94 ± 5.25^b^	5 ± 0^b^	34.25 ± 0.06^b^	8.1 ± 1.2^b^	44.6 ± 1.34^b^	51.2 ± 1.1^b^	8.1 ± 0.14^a^	4.5 ± 0.25^b^	12.6 ± 0.1^b^	1.8 ± 0.24^b^
T4	13.88 ± 0.62^ab^	94.47 ± 0.18^ab^	6 ± 0^a^	35.36 ± 0.37^a^	8.3 ± 0.9^b^	43.5 ± 0.95^b^	49.3 ± 1.2^b^	7.9 ± 0.05^a^	4.4 ± 0.23^a^	12.3 ± 0.1^b^	1.8 ± 0.15^b^
T5	14.28 ± 0.49^a^	85.11 ± 0.24^a^	3.5 ± 0^c^	27.78 ± 0.12^a^	6.5 ± 1.1^a^	40.5 ± 1.2^a^	47.6 ± 1.54^a^	7.6 ± .0.06^a^	4.3 ± 0.1^a^	11.9 ± 0.31^a^	1.76 ± 0.24^a^

*Values after (±) represents standard deviation (SD). The means with different letter differ significantly at p < 0.05 (Tukey’s HSD test).*

**TABLE 9:** Macro and micro nutrient content of *Eucalyptus Camaldulensis* plants before and after treatment.

	Na (%)	K (%)	N (%)	P (%)	Ca (%)	Mg (%)	Zn (mg/kg)	Cu (mg/kg)	Fe (mg/kg)	Mn (mg/kg)
**Leaf**										
Initial (Day 0)	0.05 ± 0.01^a^	0.08 ± 0.01^a^	0.04 ± 0^a^	0.12 ± 0^a^	0.35 ± 0^a^	0.18 ± 0^a^	12.91 ± 0.1^a^	3.71 ± 0.22^a^	128.33 ± 5.33^a^	68.72 ± 1.82^a^
**After treatment**										
T1	1.17 ± 0.02^b^	2.28 ± 0.04^b^	1.64 ± 0.06^b^	0.29 ± 0.04^b^	1.01 ± 0.03^b^	0.47 ± 0.02^b^	46.88 ± 0.65^b^	11.17 ± 0.39^b^	766.42 ± 2.48^b^	198.36 ± 3.42^b^
T2	1.39 ± 0.01^c^	2.62 ± 0.03^c^	1.67 ± 0.04^b^	0.34 ± 0.02^c^	1.19 ± 0.04^c^	0.67 ± 0.02^c^	51.89 ± 0.72^bc^	18.1 ± 0.41^c^	867.32 ± 2.75^c^	218.72 ± 2.47^b^
T3	1.57 ± 0.01^d^	2.82 ± 0.07^d^	1.69 ± 0.08^bc^	0.35 ± 0.05^c^	1.2 ± 0.05^c^	0.67 ± 0.03^c^	53.49 ± 0.62^c^	19.4 ± 0.47^d^	857.88 ± 1.99^bc^	225.07 ± 2.09^c^
T4	1.59 ± 0.02^d^	2.86 ± 0.05^d^	1.71 ± 0.07^bc^	0.36 ± 0.05^c^	1.21 ± 0.03^c^	0.68 ± 0.01^c^	53.11 ± 0.87^bc^	18.55 ± 0.31^c^	878.23 ± 3.11^c^	224.14 ± 3.11^c^
T5	1.41 ± 0.03^c^	2.76 ± 0.01^cd^	1.68 ± 0.04^c^	0.31 ± 0.03^b^	1.18 ± 0.01^c^	0.72 ± 0.05^cd^	53.14 ± 0.47^bc^	19.19 ± 0.33^d^	854.78 ± 2.82^bc^	217.49 ± 2.98^b^
**Shoot**										
Initial (Day 0)	0.01 ± 0^a^	0.13 ± 0.01^a^	0.02 ± 0a	0.04 ± 0a	0.28 ± 0.01^a^	0.1 ± 0^a^	10.42 ± 0.27^a^	5.69 ± 0.35^a^	50.07 ± 1.23^a^	62.42 ± 1.63^a^
**After treatment**										
T1	1.85 ± 0.06^b^	2.56 ± 0.04^b^	1.12 ± 0.02^b^	0.08 ± 0.02^b^	0.62 ± 0.04^b^	0.48 ± 0.05^b^	39.34 ± 0.53^b^	15.07 ± 0.31^b^	323.75 ± 2.44^b^	192.86 ± 3.38^b^
T2	2.06 ± 0.08^b^	3.23 ± 0.05^c^	1.25 ± 0.03^c^	0.12 ± 0.01^c^	0.84 ± 0.07^b^	0.55 ± 0.03^c^	45.87 ± 0.56^b^	16.74 ± 0.31^b^	414.87 ± 2.69^c^	202.93 ± 2.45^b^
T3	2.23 ± 0.07^b^	3.3 ± 0.05^c^	1.32 ± 0.01^c^	0.12 ± 0.02^c^	0.85 ± 0.05^b^	0.54 ± 0.04^c^	47.66 ± 0.48^b^	16.56 ± 0.37^b^	467.72 ± 1.97^c^	212.05 ± 2.05^b^
T4	2.24 ± 0.03^b^	3.43 ± 0.01^c^	1.29 ± 0.03^c^	0.13 ± 0.01^c^	0.86 ± 0.03^b^	0.56 ± 0.05^c^	49.23 ± 0.81^c^	17.22 ± 0.29^b^	435.85 ± 3.05^b^	208.72 ± 3.09^b^
T5	2.16 ± 0.01^b^	2.61 ± 0.03^b^	1.26 ± 0.02^c^	0.12 ± 0.04^c^	0.83 ± 0.04^b^	0.54 ± 0.02^c^	45.03 ± 0.45^b^	15.18 ± 0.27^b^	420.04 ± 2.78^b^	218.36 ± 2.9^b^
**Root**										
Initial (Day 0)	0.15 ± 0.01^a^	0.12 ± 0.01^a^	0.03 ± 0.01^a^	0.01 ± 0^a^	0.44 ± 0.03^a^	0.15 ± 0.01^a^	12.6 ± 1.03^a^	8.74 ± 0.99^a^	145.46 ± 6.31^a^	63.64 ± 2.15^a^
**After treatment**										
T1	2.03 ± 0.04^b^	1.98 ± 0.04^b^	0.54 ± 0.04^b^	0.1 ± 0.03^b^	0.53 ± 0.04^b^	0.25 ± 0.04^b^	31.06 ± 0.59^b^	11.25 ± 0.35^b^	659.32 ± 2.46^b^	209.49 ± 3.4^b^
T2	2.48 ± 0.05^c^	2.47 ± 0.04^c^	0.62 ± 0.04^b^	0.12 ± 0.02^b^	0.79 ± 0.06^c^	0.31 ± 0.03^b^	36.04 ± 0.64^b^	14.34 ± 0.36^c^	798.01 ± 2.72^c^	239.77 ± 2.46^c^
T3	2.5 ± 0.04^c^	2.53 ± 0.06^c^	0.65 ± 0.05^b^	0.12 ± 0.04^b^	0.78 ± 0.05^c^	0.31 ± 0.04^b^	35.63 ± 0.55^b^	13.98 ± 0.42^c^	752.97 ± 1.98^c^	221.96 ± 2.07^c^
T4	2.56 ± 0.03^c^	2.75 ± 0.03^d^	0.67 ± 0.05^b^	0.13 ± 0.03^b^	0.75 ± 0.03^c^	0.31 ± 0.03^b^	37.09 ± 0.84^b^	15.69 ± 0.3^c^	769.7 ± 3.08^c^	227.82 ± 3.1^c^
T5	2.38 ± 0.02^c^	2.65 ± 0.02^d^	0.63 ± 0.03^b^	0.13 ± 0.04^b^	0.82 ± 0.03^c^	0.32 ± 0.04^b^	37.07 ± 0.46^b^	14.75 ± 0.3^c^	820.81 ± 2.8^d^	224.28 ± 2.94^c^

*Values after (±) represents standard deviation (SD). The means with different letter differ significantly at p < 0.05 (Tukey’s HSD test).*

The height and the root length of the plant were 30 and 8 cm at the start of the experiment. Enhanced heights were obtained at the end and were highest in T3 and T4 treatments. The average percentage increase in plant height was 194; 39%, where the highest increase was in T4 (213.6%) followed by T3 (212.09%), T5 (182.5%), T2 (197.6%), and T1 (166%). Likewise, a similar trend of growth rate was observed in root length. Percentage increase in root length was as follows: 342% (T4), 328% (T3), 266% (T2), 247% (T5), and 241% (T1). Similar results are also reported by Keshtkar et al. (2016). Plant height and number of new shoots show similar values as compared with the findings of Coung et al. (2015); however, the root length was higher in our study (above 80 cm) in all the treatments in comparison with the study of Cuong et al. (2015), i.e., only 10 cm. The chlorophyll content of the plants grown in different treatments was not significantly different (*p* > 0.05) except for T1, which has the least value. The total chlorophyll content was highest in T3 (12.6 mg/L) followed by T4 (12.3 mg/L), T5 (11.9 mg/L), T2 (11.5 mg/L), and control, that is, T1 (11.0 mg/L). Our results were compared with the study of Akhzari et al. (2013), and similar ranges of chlorophyll content were found; however, the selected plant species was different.

Greater growth and biomass production in Eucalyptus plants were also evident in all treatments except for the control, which may be due to sufficient availability of water and essential elements adsorbed at the FBM (Bhati and Singh, 2003). Root and shoot dry weight (biomass) were highest in T3 (51.2 and 44.6 g) and T4 (49.3 and 43.5 g) followed by T5 (47.6 and 40.5 g), T2 (46.9 and 38.2 g), and T1 (45.6 and 37.1 g). Similarly, higher concentrations of essential micronutrients in treatments could be due to the increasing solubility of metal ions resulting in enhanced growth of the plant (Bhati and Singh, 2003). Enhanced growth of *Eucalyptus camaldulensis* is also shown in the study of Shah et al. (2010) in which a pot experiment was conducted, and the effect of industrial effluent was investigated on the growth and element accumulation by *Eucalyptus camaldulensis.* The plant irrigated with wastewater showed increased stem height, biomass, leaf count, and dry weights. Nutrient accumulation was also higher in wastewater-irrigated plants. Similar results are also found in the present study. Another study concludes that Eucalyptus shows better performance in terms of survival, biomass, and growth among different plant species when irrigated with saline wastewater (Tomar et al., 2003). Similar conclusions are also made in the studies of Nawaz et al. (2016) and Zouari et al. (2020). Therefore, based on the findings of the present study, it may be suggested that irrigation of plant species with wastewater offers a better option and further can be useful as an optimal strategy for raising woodlot to supply fuel for people. Moreover, to identify the role of FBM in plants, it is concluded that FBM acts as a buffer, promoting substantial absorption of nutrients by plants ensuring no TDS or salt-related toxicity.

Studies using a combination of organic raw material and forestry tree species are very limited and include mainly the high-rate transpiration system (HRTS)-based treatment of wastewater. One such study by Yadav et al. (2010) employs the HRTS system for the treatment of wastewater from the paper and pulp industry, and it showed significant removal of salts, BOD, and COD. The experiment included the use of filter media consisting of sawdust, fly ash, eucalyptus leaves, and lime sludge and gypsum, which was applied in furrows, and plantation of plants such as *Dendrocalamus strictus, Bambusa arundinacea, Azadirachta indica, Dalbergia sissoo, Eucalyptus hybrid* and *Eucalyptus robusta, Tectona grandis* and *Cassia siamea* in ridges. A similar study by Singh et al. (2008) shows high TDS, COD, and BOD removal (80–100%) from distillery effluent, which was used for irrigation of plant species *Dendrocalamus strictus* in a lysimeter study along with coconut husk as the filter media. Significant TDS treatments were also obtained for pulp and paper mill wastewater using a mixture of gypsum, coconut husk, bamboo dust, and sawdust as filter media and *Dendrocalamus strictus, Bambusa arundinacea, Azadirachta indica, Dalbergia sissoo, Eucalyptus hybrid* and *Eucalyptus robusta, Tectona grandis* and *Cassia siamea* as plant species (Thawale et al., 2015). Such encouraging results from these limited studies determines the feasibility of land-based treatment of a variety of wastewater using a combination of forestry tree species having high salt tolerance capacity and FBM.

## Conclusion and Recommendation

Based upon outcomes of the study, it can be concluded that the relative capacity of rice and coconut husk as bedding material for TDS management is highest among all other treatments. However, other bio-adsorbents, such as rice straw and charcoal, can also be effectively utilized for treatment purposes based on their local availability. The salt reduction from wastewater was also very high (60–70%) because of the adsorption of salts on FBM. Consequently, about 80–88% of total leachate volume reduction was obtained in the treatments. Therefore, this approach can efficiently be modified to a large scale, such as constructed wetland, to fulfill the regulatory need of zero discharge from industries. The soil after application showed no saline or sodic conditions because of the presence of FBM in treatment systems, thereby maintaining the physicochemical and biological integrity of the soil. Therefore, it was inferred that FBM not only plays a significant role in management of TDS but also favors plant growth. The plant growth data confirms the role of FBM asa buffer not allowing the roots to experience toxicity due to Na^+^/EC/TDS. However, further studies are needed that can emphasize the use of a proper combination of FBM for different wastewater treatment by extending the knowledge generated in this study. In this study, *Eucalyptus* plants accumulated Na, Ca, Mg, and K and higher biomass with no adverse impact on the growth as a function of wastewater, which indicates that sal- tolerant plant species can be utilized in the treatment of wastewater. The absolute combination of forestry plant species and organic raw material showed the feasibility of treatment of wastewater with very high TDS (up to 600 mg/L). The study can be further extended to other salt-tolerant plant species, such as *Prosopis, Acacia, Casuarina*, etc. in the field conditions in combination with other organic raw materials to further enhance the treatment and TDS management efficiency of the lysimeters or land-based treatment systems. Such approach can also be applied to restore the productivity of degraded lands by saline wastewater irrigation. Furthermore, utilization of organic raw materials such as coconut husk, rice husk, and rice straw as FBMs for saline wastewater management might offer socioeconomic solutions and sustainable management options of these organic residues, which are generated in huge quantities and have various environmental implications.

## Data Availability Statement

The original contributions presented in the study are included in the article/supplementary material, further inquiries can be directed to the corresponding author/s.

## Author Contributions

DM has prepared the first draft of the manuscript, performed the experiments, and compiled the results, and also performed data interpretation. KR helped in the finalization of data and data interpretation and final draft preparation. AS reviewed the manuscript for grammatical and language errors and helped in preparation of graphs and performed the statistical interpretations. KK and PT designed the experiments, performed data analysis, and provided editorial advice in the manuscript and supervised the work.

## Conflict of Interest

The authors declare that the research was conducted in the absence of any commercial or financial relationships that could be construed as a potential conflict of interest.

## Publisher’s Note

All claims expressed in this article are solely those of the authors and do not necessarily represent those of their affiliated organizations, or those of the publisher, the editors and the reviewers. Any product that may be evaluated in this article, or claim that may be made by its manufacturer, is not guaranteed or endorsed by the publisher.
